# Intraoperative 3D fluoroscopy accurately predicts final electrode position in deep brain stimulation surgery

**DOI:** 10.1007/s00701-024-06214-8

**Published:** 2024-08-07

**Authors:** Patrícia Neto-Fernandes, Clara Chamadoira, Carolina Silva, Leila Pereira, Rui Vaz, Manuel Rito, Manuel J. Ferreira-Pinto

**Affiliations:** 1 Faculty of Health Sciences, University of Beira Interior, Covilhã, Portugal; 2 Department of Neurosurgery, Centro Hospitalar Universitário de São João, Porto, Portugal; 3 Department of Clinical Neurosciences and Mental Health, Faculty of Medicine, University of Porto, Porto, Portugal; 4 Department of Radiology, Centro Hospitalar Universitário de São João, Porto, Portugal; 5 Department of Surgery and Physiology, Faculty of Medicine, University of Porto, Porto, Portugal

**Keywords:** Intraoperative 3D fluoroscopy, Deep brain stimulation, Stereotactic surgery, Electrode location

## Abstract

**Purpose:**

In the absence of an intraoperative CT or MRI setup, post-implantation confirmation of electrode position in deep brain stimulation (DBS) requires patient transportation to the radiology unit, prolonging surgery time. This project aims to validate intraoperative 3D fluoroscopy (3DF), a widely available tool in Neurosurgical units, as a method to determine final electrode position.

**Methods:**

We performed a retrospective study including 64 patients (124 electrodes) who underwent DBS at our institution. Intraoperative 3DF after electrode implantation and postoperative volumetric CT were acquired. The Euclidean coordinates of the electrode tip displayed in both imaging modalities were determined and inter-method deviations were assessed. Pneumocephalus was quantified and its potential impact in determining the electrode position analyzed. Finally, 3DF and CT-imposed exposure to radiation was compared.

**Results:**

The difference between the electrode tip estimated by 3DF and CT was 0.85 ± 0.03 mm, and not significantly different (*p* = 0.11 for the distance to MCP assessed by both methods), but was, instead, highly correlated (*p* = 0.91; *p* < 0.0001). Even though pneumocephalus was larger in 3DF (6.89 ± 1.76 vs 5.18 ± 1.37 mm^3^ in the CT group, *p* < 0.001), it was not correlated with the difference in electrode position measured by both techniques (*p* = 0.17; *p* = 0.06). Radiation exposure from 3DF is significantly lower than CT (0.36 ± 0.03 vs 2.08 ± 0.05 mSv; *p* < 0.0001).

**Conclusions:**

Intraoperative 3DF is comparable to CT in determining the final DBS electrode position. Being a method with fewer radiation exposure, less expensive, faster and that avoids patient transportation outside the operation room, it is a valid tool to replace postoperative CT.

## Introduction

Deep brain stimulation (DBS) is a well-established treatment for movement disorders such as Parkinson’s disease (PD), essential tremor, dystonia, as well as refractory epilepsy and obsessive–compulsive disorder [6, 7]. This procedure entails the implantation of electrodes in specific brain areas, which deliver adjustable electrical impulses in order to modulate the activity of neuronal circuits and provide therapeutic benefits. While DBS does not cure the underlying condition, it can significantly improve the quality of life of patients [11]. In PD, for example, it has established itself as an effective treatment to improve symptoms such as tremor, rigidity and bradykinesia [5].

The efficacy of DBS critically depends on the exact electrode position. Given the small dimensions of the targeted nuclei, the therapeutic effect relies on the accurate modulation of the intended target and avoidance of unintended nearby structures, which could elicit side effects [6]. In order to fine-tune the final electrode location, multiple intraoperative techniques are employed, such as microelectrode recording of neuronal activity and microstimulation trials [8]. However, anatomical confirmation of the true electrode location post-implantation is crucial to exclude unwanted displacement during implantation of the definitive electrode [16].

Multiple imaging techniques have been described for this end, namely two-dimensional fluoroscopy, a method that is easily available in the operating room (OR), but offers limited accuracy, given that it is not three-dimensional. Alternatively, three-dimensional methods such as computed tomography (CT) and magnetic resonance imaging (MRI) can be employed, but they imply either ORs with integrated CT/MRI setups [1–3, 17] (which are highly-expensive facilities, only available in a few centers worldwide) or patient transportation to the radiology unit during surgery. Although the latter is the most commonly employed method, transporting the patient, often under general anaesthesia, out of the OR can pose safety issues and significantly increases operative time and associated costs [5, 13, 16].

In this context, intraoperative three-dimensional fluoroscopy (3DF) with a C-arm has been proposed as a fast, inexpensive alternative, readily available in the OR and requiring lower radiation exposure compared to CT [9, 15, 16, 19]. Recent studies, with small patient samples, suggest a possible usefulness of 3DF in estimating the final position of electrodes in stereotactic procedures such as DBS and stereoelectroencephalography [4, 5, 16, 18, 19]. However, no study has directly compared the two methods employed in the same patient across a large number of patients/electrodes. Instead some studies compare different cohorts of patients undergoing either CT or 3DF. In order to effectively assess the accuracy of 3DF in relation to that of CT, we believe that it is essential to determine final electrode location using both methods in the same patient, and across a large number of patients.

In this context, the current work aims to evaluate the accuracy of 3DF in estimating the final position of electrodes implanted in DBS surgery, taking as a reference the location determined by CT, the standard method at our department. A large cohort of 124 electrodes from 64 patients was included and 3DF and CT images were acquired from every patient. We believe our findings validate 3DF as an effective tool to determine the final electrode position in DBS. Given that it is faster, cheaper, readily available in the OR and poses lower radiation exposure, it may replace the currently standard intraoperative CT.

## Methods

### Patient selection

This is a retrospective study that includes a cohort of patients who underwent DBS surgery at our department from May 2019 to January 2022. Seventy patients who received intraoperative 3DF and early postoperative CT (within 48 h of surgery) were initially selected to be included. After applying our exclusion criteria, low-resolution CT scan (> 2 mm slice width) and need for revision surgery between both imaging methods), 4 patients were excluded. Two additional patients were excluded as it was not technically possible to retrieve their 3DF images from the electronic records. Therefore, 64 patients and a total of 124 implanted electrodes were included in the analysis: 58 patients with Parkinson’s disease who were implanted bilaterally in the subthalamic nucleus, 4 patients with chronic pain who were implanted unilaterally in the left ventral posterolateral thalamic nucleus, 1 patient with refractory epilepsy implanted bilaterally in the anterior nucleus of the thalamus, 1 patient with dystonia implanted bilaterally in the globus pallidus internus.

### Surgical procedure and imaging

All patients underwent frame-based stereotactic implantation of electrodes for DBS using the Leksell G frame. Briefly, after securing the stereotactic frame to the patient’s head, a pre-implantation stereotactic CT was acquired and fused with a preoperative MRI, which was used to plan the electrode trajectory and target. After extracting the stereotactic coordinates, definitive electrodes were implanted in the desired target and secured to the skull. After the cranial skin was sutured, a fluoroscopic image acquisition and 3D reconstruction with the C-arm system (Ziehm^®^) was performed intraoperatively, and, finally, the pulse generator was implanted in a subcutaneous pocket created in the subclavicular region. A postoperative high-resolution CT scan (1 mm slice width) was acquired within the first 48 h after surgery.

**Fig. 1:**
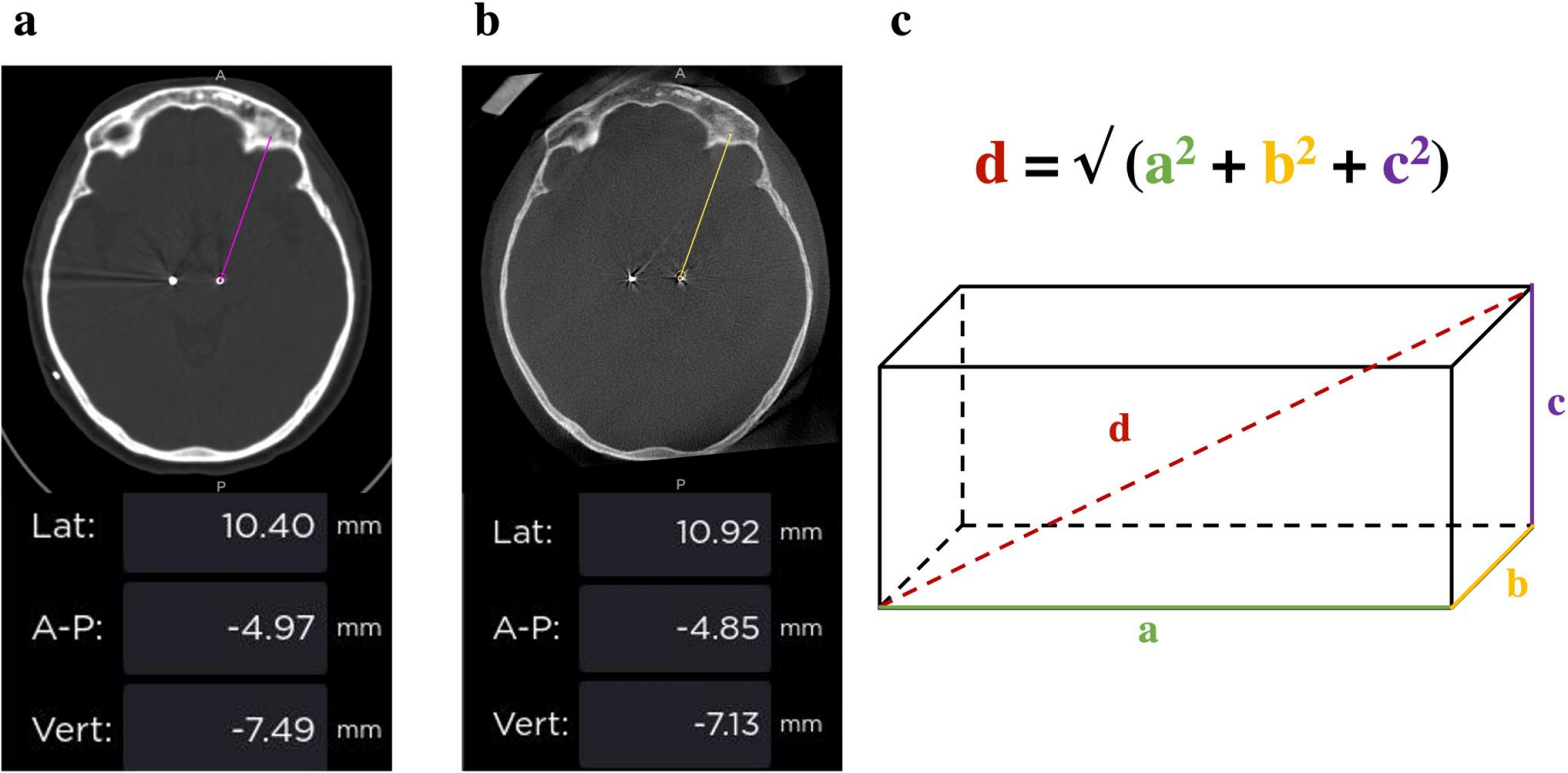
Electrode tip identification and distance calculation. **a** Detection of the electrode tip on CT and respective Euclidean coordinates; **b** the same electrode tip is detected on 3DF and its Euclidean coordinates are extracted; **c** using the respective x, y and z coordinates, the distance between both points (3DF-CT distance) is determined with the Pythagorean theorem applied to space

In order to compare the accurary of 3DF to that of CT in determining the final electrode position, the following methodology was employed. Intraoperative 3DF, early postoperative CT and preoperative MRI scans (high resolution - 1 mm slice width - T1 + gadolinium) were merged in a stereotactic planning station (Medtronic StealthStation S8^®^). The midcommissural point (MCP) was determined in the MRI images and used as a reference for the Euclidean coordinates. Then, the spatial position of the electrode tip was manually determined in the 3DF and CT images, independently, and the respective Euclidean coordinates were extracted (Fig. 1a). Assuming the position of the electrode in CT as a reference, its position in 3DF and consequent distance between both will allow us to quantify the difference between both techniques. In order to calculate this distance, we used the xyz coordinates (representing the medio-lateral, anterior–posterior and dorso-ventral coordinates, respectively) of the electrode tip in 3DF (x1, y1 and z1) and CT (x2, y2 and z2) and employed the Pythagorean theorem applied to space (Fig. 1b), as follows:

$$\small 3\text{DF}-\text{CT distance}=\surd ((\text{x}1-\text{x}2)^2 + (\text{y}1-\text{y}2)^2 + (\text{z}1-\text{z}2)^2)$$

Furthermore, in order to statistical probe the significance of the difference between the two methods, the distance of the electrode tip to MCP (whose coordinates are x = 0, y = 0 and z = 0 by definition) in both imaging methods was determined following analogous formulas:

$$\begin{array}{c}3\text{DF}-\text{MCP distance }= \surd ((\text{x}1)^2 +(\text{y}1)^2 +(\text{z}1)^2)\\ \text{CT}-\text{MCP distance }= \surd ((\text{x}2)^2 +(\text{y}2)^2 +(\text{z}2)^2)\end{array}$$

### Pneumocephalus analysis

Given that the postoperative CT scan was acquired up to 48 h after surgery, the time gap with the intraoperative 3DF was a concern. Furthermore, the difference in head position between modalities (head-fixed in the stereotaxic frame, in slight flexion during acquisition of intraoperative 3DF and head-free in supine position during postoperative CT acquisition) could also be a potential source of mismatch between both methods, if brain shift was a significant factor. Specifically, pneumocephalus, the presence of intracranial air, is a common DBS-related phenomenon which can lead to brain shift and targeting inaccuracy [10].

In order to control for this potential issue, we performed 3D reconstructions of pneumocephalus in our 3DF and CT images and quantified their volume. Furthermore, a correlation analysis was performed in order to assess whether the difference between 3DF and CT pneumocephalus was related to the magnitude of 3DF-CT electrode distance.

### Radiation exposure analysis

In order to compare radiation exposure imposed by both imaging methods, data was extracted from dose reports provided by both devices. Given that delivered radiation dose was provided as dose area product (DAP) in mGy.cm^2^ in 3DF reports and as dose length product (DLP) in mGy.cm in CT reports, and that these factors cannot be directly compared or interconverted, we converted both datasets into effective dose (ED) exposure in mSv, in order to correctly compare both methods. In line with the reported literature [9, 19], conversion factors used for DAP to ED and DLP to ED were 0.091 mSv.Gy^−1^.cm^−2^ and 0.0021 mSv.mGy^−1^.cm^−1^, respectively.

### Statistical analysis

Data are displayed as mean ± standard error of mean and significance level was set to *p* < 0.05. Statistical analysis and plots were performed using Graphpad Prism Version 8^®^ software. Datasets were probed for normality using the D’Agostino-Pearson Test. As our datasets failed to pass the normality test, non-parametric Wilcoxon signed rank test was employed to compare paired samples and Spearman’s correlation coefficient (ρ) was calculated to assess the correlation between two variables.

## Results

### 3DF accurately predicts electrode tip position

A total of 124 electrodes, from 64 patients, were analyzed and their tip position was assessed in 3DF and CT images.

**Fig. 2:**
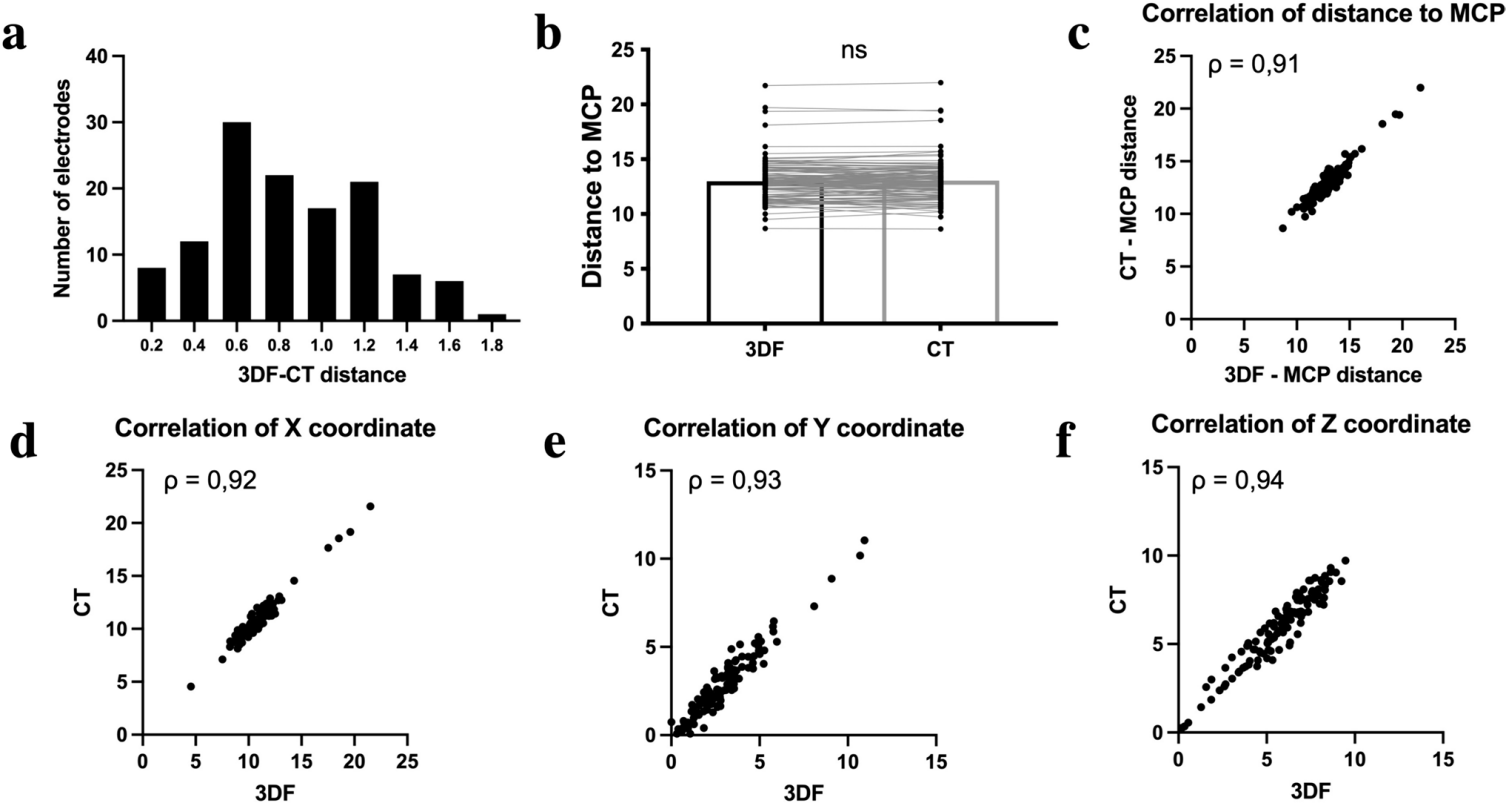
Electrode tip metrics. **a** histogram depicting the distribution of 3DF-CT distances; **b** distance to MCP in both imaging methods; each dot represents one individual electrode; horizontal lines connect the same electrode measured in 3DF and CT; **C**-**F**) Spearman’s correlations across both groups of: **c** distance to MCP (ρ = 0.91; *p* < 0.0001); **d** x coordinate value (ρ = 0.92; *p* < 0.0001); **e** Y coordinate value (ρ = 0.93; *p* < 0.0001); **f** Z coordinate value (ρ = 0.94; *p* < 0.0001); ns = *p* > 0.05; distances and coordinate values are in mm

The distance between electrode tip position, measured in 3DF and CT imagens was 0.85 ± 0.03 mm, which is within the resolution limit of the CT scans, which had a slice width of 1 mm. Distribution of values is displayed in Fig. 2a; 65% of analyzed electrodes had 3DF-CT deviations smaller than 1 mm; 94% smaller than 1.5 mm and 100% below 2 mm.

In order to test the statistical significance of this deviation, we compared the distance of the electrode tip to the MCP, as measured by the two methods (Fig. 2b). The electrode tip distance to MCP was 13.00 ± 0.16 mm in the 3DF group and 13.06 ± 0.16 mm in the CT group, and were not significantly different (*p* = 0.11). Furthermore, these variables are strongly correlated (ρ = 0.91; *p* < 0.0001; Fig. 2c). In agreement with this, all electrode tip x, y and z coordinates are highly correlated between both methods, suggesting that there is no bias towards a specific axis in the deviation between both methods (for x: ρ = 0.92; *p* < 0.0001; for y: ρ = 0.93; *p* < 0.0001 and for z: ρ = 0.94; *p* < 0.0001; Fig. 2d, e and f). Considering these findings altogether, we conclude that the accuracy of the 3DF in estimating electrode position is comparable to that of CT.

### Brain shift does not impact the 3DF – CT comparison

**Fig. 3:**
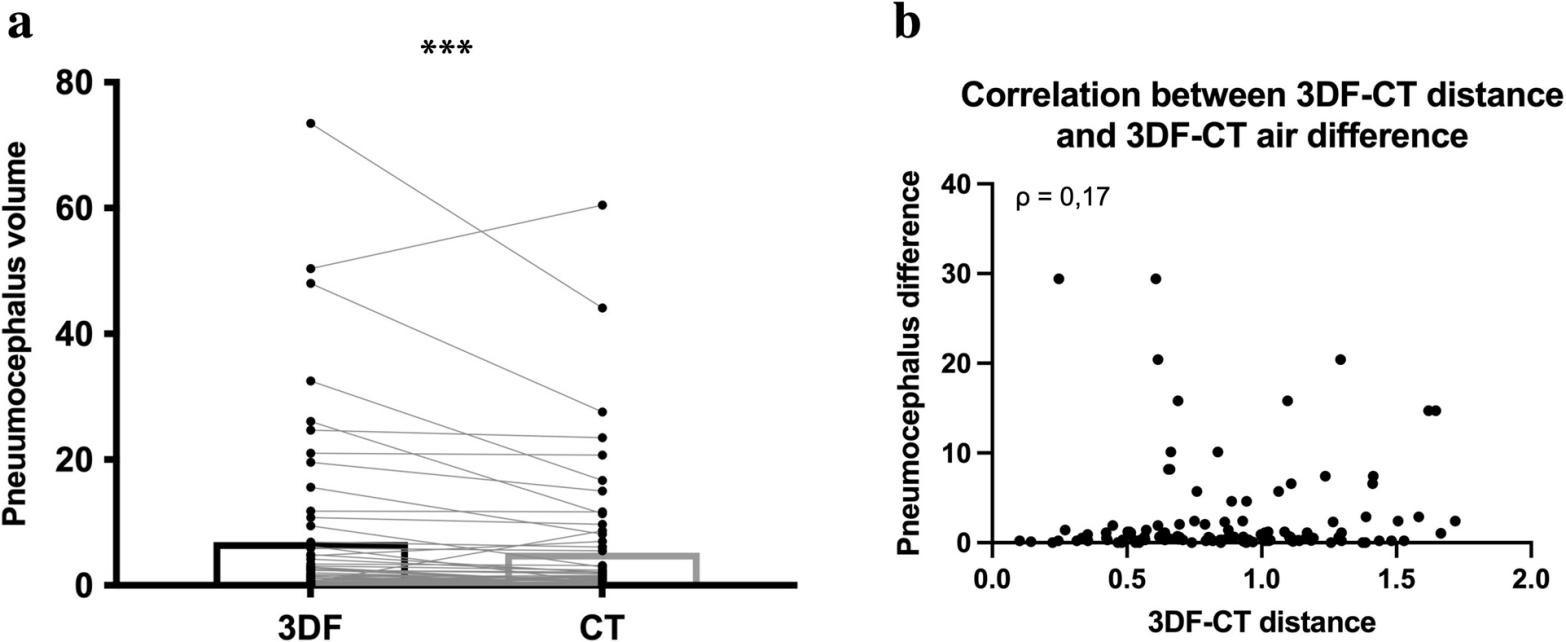
Pneumocephalus evaluation. **a** Pneumocephalus volume was significantly higher in the 3DF images, when compared to CT images (*p* = 0.0007); **b** however, there was no correlation between the pneumocephalus volume difference in 3DF and CT images and electrode tip 3DF-CT distance (ρ = 0.17; *p* = 0.064); *** = *p* < 0.001; volumes are displayed in mm^3^ and distances in mm

As previously mentioned, potential brain shift between the timing of acquisition of both imaging methods was a concern. In order to assess whether this phenomenon was impacting our electrode tip position results, we quantified the volume of pneumocephalus in 3DF and CT images of 61 patients (three patients could not be included in this analysis because the 3DF images did not cover the most frontal part of the cranium, precluding pneumocephalus measurement). In fact, the volume of intracranial air was significantly higher in the 3DF images than the CT counterparts (6.89 ± 1.76 and 5.18 ± 1.37 mm^3^, respectively; *p* = 0.0007; Fig. 3a). This difference can be explained by the fact that the 3DF is acquired shortly after dura-mater opening during surgery, while our CT data pertains to up to 48 h after surgery, when it is expected that some of the intracranial air was already reabsorbed. However, this pneumocephalus values are rather small, when considering the total intracranial space and may not necessarily cause a relevant shift of the targeted deep nuclei and, consequently the electrode tip position across both methods.

In order to probe this, we postulated the following rational: if pneumocephalus volume difference across both methods is impacting electrode tip position, then patients with higher pneumocephalus difference between 3DF and CT would also have higher distances between electrode tip as detected in both methods. Therefore, we performed a Spearman correlation analysis and found no significant correlation between pneumocephalus difference and electrode tip distance across both methods (correlation coefficient of 0.17; *p* = 0.064; Fig. 3b). Therefore, we conclude that the difference in intracranial air detected in both imaging methods did not impact the distance between electrode tip measured in 3DF and CT images.

### 3DF entails significantly lower effective radiation dose exposure

**Fig. 4:**
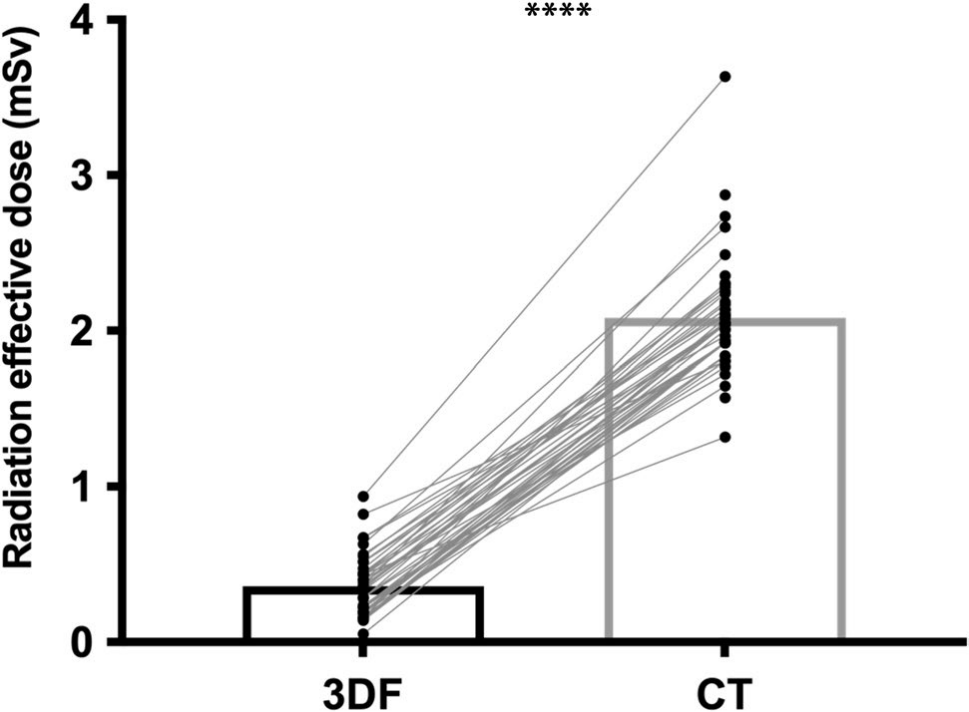
Radiation exposure from 3DF and CT. Effective-dose radiation exposure was significantly lower in 3DF, when compared to CT (*p* < 0.0001); **** = *p* < 0.0001

As expected, after converting delivered radiation dose (DAP and DLP) from both modalities to ED, the effective dose exposure imposed by 3DF was significantly lower than the one from CT (0.36 ± 0.03 vs 2.08 ± 0.05 mSv; *p* < 0.0001, Fig. 4). In light of these values, radiation exposure from 3DF is only 17% of that of CT, which is not only a statistically, but also clinically significant reduction.

## Discussion

Correct lead position in DBS is absolutely crucial for the success of the procedure, as it has been correlated with improved clinical outcomes. For example, a 2 mm deviation in subthalamic nucleus electrodes may produce an inadequate outcome [12]. Therefore, imaging methods to determine the final electrode position must produce accurate anatomical information. With this in mind, the main goal of the present study was to assess whether 3DF has comparable accuracy to CT in predicting final electrode position in DBS surgery. In order to do this, we quantified the distance between the electrode position estimated by 3DF and CT in the same patients and seek to understand whether that difference was clinically meaningful. We were able to include a large number of patients and electrodes in this analysis, which, to our knowledge, makes it the largest sample size in studies addressing this research question. Another important strength is that we directly compare both methods across the exact same electrodes implanted in the exact same patients, unlike other studies which compare these imaging modalities across two separate groups of patients. In order to produce strong evidence of equality of these modalities, they must be employed to directly compare the position of the same electrodes of the same patients. Therefore, we believe that this study design significantly empowers the magnitude of our findings.

We observed that the inter-method difference was 0.85 mm and conclude that it is not clinically relevant, for the following reasons. First and foremost, this difference is well within the limits of our methodology resolution which are mostly imposed by CT resolution (slice width of 1 mm), but also by the volume of the metallic artifact of the electrodes which can be a source of infra-millimetric variability when assigning the electrode tip. Furthermore, if a specific systematic error occurred when estimating the distance between methods, it would likely occur in a specific direction, affecting one Euclidean coordinate more than the others. Our data shows that all coordinates are highly correlated between both methods and, therefore, this small variation between 3DF- and CT-estimated electrode position is stochastic. Finally, statistical comparison between distance of electrode tip to the spatial reference – the MCP – did not show a significant difference between both methods. Taken together, these findings strongly support that 3DF is comparable to CT in assessing DBS electrode location.

Another important aspect to consider is that our 3DF scans were performed intraoperatively, while CT scans were conducted within the first 48 h after surgery. Consequently, any potential procedure-related brain shift could have influenced the electrode position in 3DF and CT to different degrees, as intracranial air might have partially been reabsorbed within 48 h [14, 19]. While pneumocephalus has been postulated to cause brain shift during DBS [17], some evidence suggests that brain shift might affect mostly superficial targets and not deep-seated regions such as the basal ganglia [10]. In line with these concepts, we observed that pneumocephalus volume was higher in the intraoperatively acquired 3DF than the postoperative CT scan. However, there was no correlation between the degree of pneumocephalus difference across methods and the degree of distance between the electrode tip estimation by both methods. If pneumocephalus difference between both methods influenced the electrode tip location, one would predict that patients with higher intracranial air differences between both images would also display a higher degree of difference between electrode tip position across both methods. Therefore, we conclude that the time gap between the acquisition of both imaging studies is unlikely to influence our ability to compare them.

Our findings are comparable to those already published in the literature. In a 2014 study, Weise et al. concluded that the accuracy of 3DF imaging in phantoms revealed a slightly lower accuracy but higher precision than the CT [19]. Furthermore, in a 2020 study, 3DF-based frame registration showed similar implantation accuracy to CT imaging [4]. Finally, a 2021 study by Restrepo et al. concluded that the use of 3DF as a method for registration resulted in similar implantation accuracy compared with CT [16].

Another important point to discuss when comparing 3DF with CT methodologies is radiation exposure. In line with our findings, multiple studies have also described a clear benefit of 3DF over CT in this regard. The above mentioned Weise 2014 study concluded that the effective dose for 3DF was 0.65 mSv, while that for CT is almost double [19]. Additional studies comparing radiation exposure from both methods in DBS and SEEG procedures also estimated that 3DF radiation dose was about 20% of that of CT [4, 16], which is a value in line with our present findings.

When considering replacing intraoperative post-implantation CT with 3DF, one important question must be considered. Unlike CT, 3DF does not image soft tissues and, therefore, cannot assess for implantation-induced hemorrhage. Nevertheless, we believe that this is not a relevant caveat of 3DF, given that, if rulling out a hemorrhage is deemed important, patients can still undergo a postoperative CT scan after leaving the operation room or the day after. Furthermore, this CT scan can be of low resolution (and, therefore, entail dramatically lower radiation dose), given that it is not meant to probe electrode position, which was already assessed with intraoperative 3DF. Additionally, a clinically relevant hemorrhage would likely entail an intraoperative hemodynamic event or an intraoperative or postoperative neurological sign. In the absence of both, a hemorrhagic event requiring intervention is unlikely.

### Study limitations

There are some limitations to our study, which have partially been addressed in previous sections. One important point is the difference in time and settings of acquisition of both imaging modalities. Also, the resolution limitations inherent to our methodologies, namely the metallic electrode artifact, the imaging studies resolution and the manual determination of the electrode tip. We believe that the above-mentioned strengths of our study significantly prevent these limitations from impairing our conclusions. Despite them, we still found an infra-millimetric difference between the 3DF and CT which is not clinically relevant. We believe that these limitations are likely preventing us from finding even smaller differences between methods rather than the other way around.

## Conclusion

Intraoperative 3DF accurately predicts the final electrode position estimated by CT. Having the added advantages of being faster, less expensive, imposing less radiation exposure and not requiring the patient to be transported outside the operation room, we believe that this method can replace CT as a post-implantation method to estimate final electrode position, which can be especially relevant in centers where intra-operation room CT or MRI is unavailable.

## Data Availability

All data supporting the findings of this study is available from the corresponding author upon reasonable request.
